# Effects of Substituting Cassava Pulp with Broken Rice and Cassava Chips in Crossbred Holstein Diets: Rumen Fermentation, Enteric Methane Emission, and Energy Utilization

**DOI:** 10.3390/ani14152257

**Published:** 2024-08-03

**Authors:** Jiraporn Kabsuk, Jenwit Nusri-un, Bhoowadol Binsulong, Thidarat Gunha, Kritapon Sommart

**Affiliations:** 1 Department of Animal Science, Faculty of Agriculture, Khon Kaen University, Khon Kaen 40002, Thailand; ka_jiraporn@kkumail.com (J.K.); jenwit.nu@kkumail.com (J.N.-u.); b.bhoowadol@kkumail.com (B.B.); 2 Department of Agricultural Management Technology, Faculty of Agricultural Technology, Valaya Alongkorn Rajabhat University under the Royal Patronage Pathum Thani Province, Pathumthani 13180, Thailand; thidarat.gunha@kkumail.com

**Keywords:** *Manihot esculenta*, *Oryza sativa*, silage, zebu, digestibility, methane

## Abstract

**Simple Summary:**

Global livestock production systems face feed availability and cost challenges, particularly for high-quality fattening beef cattle. Thus, we require research on grain-rich energy alternatives in their diets. Our study investigated the impact of substituting cassava pulp with broken rice and cassava chips in crossbred Holstein steer diets and the practical implications on the ruminant feeding system. The findings indicate that while broken rice is a superior nutrient compared to cassava pulp and chips, it significantly increased the feed costs. However, substituting cassava pulp and chips positively influenced the ensilage quality and microbial populations without adverse effects on feed intake, enteric methane emissions, and energy utilization. These results suggest that broken rice, an agro-industrial by-product, could be an efficient alternative grain-rich feed resource for ruminants. This study highlights the potential benefits of strategic feeding strategies using broken rice, particularly in tropical cattle production systems.

**Abstract:**

This study evaluates the effects of substituting cassava pulp with broken rice and cassava chips in the total mixed ration silage diets of beef cattle on feed composition, ensiling quality, digestibility, and energy utilization. Fifteen Holstein Thai native crossbred (89% *Bos taurus* × 11% *Bos indicus*) steers in the fattening phase, with an average age of 2.5 ± 0.1 years and an initial body weight of 603.7 ± 14.3 kg, were used in the energy balance trial. Using a randomized complete block design with five replications, the steers received one of three treatments. The three dietary treatments included substituting cassava pulp with cassava chips and broken rice on a dry matter basis with ratios of 50:0:0, 30:20:0, or 10:20:20. The results show that broken rice is a superior nutrient source and provides greater energy balance (*p* < 0.01). Despite the cost implications, substituting cassava pulp and chips positively impacts the ensilage pH and reduces the acetic acid concentration (*p* < 0.01). There was an increase in the lactic acid bacteria count (*p* < 0.05) and a reduction in the rumen ammonia, propionate, and butyrate concentrations (*p* < 0.05) without adverse effects (*p* > 0.05) on digestibility, blood metabolites, or enteric methane emissions. These findings suggest that broken rice is a promising alternative grain-rich ruminant feed. Future research should explore on-farm long-term feeding and economic evaluations to provide a more comprehensive understanding of the practical implications.

## 1. Introduction

At present, the beef cattle sector faces issues regarding feed cost, the environment, antibiotic use and resistance, and animal welfare [1]. Beef production systems face feed availability and cost challenges, particularly for high-yield fattening beef cattle that require a high grain or starch content in their diets for energy. This issue of global food, feed, and fuel competition is a pressing concern. However, alternative agro-industrial by-product feed sources, such as cassava pulp, promise to reduce costs and improve productivity and environmental sustainability, as demonstrated by previous research [2,3,4]. This presents an optimistic outlook for the future of livestock production, where sustainable feed sources can play a significant role.

In the search for alternative nutritional feed resources, cassava (*Manihot esculenta* Crantz) is a tropical and subtropical tuber crop that grows in poor soils and adverse climatic conditions [2,3,4,5]. Thailand’s 10% share of the world’s cassava is used to produce approximately 330 million tons of food, starch, animal feed, bioethanol, and other bioproducts yearly [6]. Approximately 54% of cassava tuber production is used for starch, 44% is used for animal feed and exports, and 2% is used for ethanol production in Thailand [6]. The efficient utilization of feed resources is essential for sustainability and cost-effectiveness. Previous research has highlighted the advantages of diets based on cassava pulp, which is a cassava starch extract by-product, such as their year-round availability, high digestibility [2,7,8], reduced enteric methane emissions [2], and ability to improve daily weight gain in beef cattle [2,8]. However, they have low protein and high fiber and moisture contents; therefore, they are perishable and have an environmental impact. Further studies on cassava pulp consumption by finishing beef cattle would provide significant and valuable insights into enhancing ruminant productivity and economic viability in tropical agriculture. On the other hand, rice grains (*Oryza sativa* L.; a long-grained variety) and cassava chips are also well-known conventional energy feeds used in diets for their superior nutrients and metabolizable energy, but they are often used in human food, pig–poultry feed, and bioethanol and have a higher price tag [9,10,11]. Compared with cassava chips, broken rice by-products from rice mills are a suitable feed resource for improved rumen digestibility because they contain more fermentable carbohydrates, protein, and fat [10,11,12]. Scheibler et al. [13] found that replacing corn with rice in 0%, 33%, 63.67%, and 100% of dairy cow diets had no adverse effect on the animals’ health, feed intake, digestibility, or milk production. Several studies reported that reducing imported feed costs by substituting rice grain with corn grain in the diets of beef and dairy cattle had no significant effect on nutrient intake and production performance [13,14,15,16]. However, there is a significant gap in the research on the effects of using cassava pulp as an alternative feed to broken rice and cassava chips in fattening beef cattle. Recently, these conventional feed costs have exponentially increased by up to 30% because of factors such as war, climate change, the bio-ethanol industry, and the increased demand for food and livestock farming.

Therefore, this study aims to assess the effects of substituting cassava pulp with broken rice and cassava chips in fattening Holstein crossbred steer diets. The research hypothesis was that this would modify the feed composition, ensiling quality, ruminal characteristics, and energy utilization in the fattened beef cattle, thereby providing insights into the potential of using broken rice as an alternative feed resource in the tropics.

## 2. Materials and Methods

### 2.1. Animals, Experimental Design, and Dietary Treatments

The Animal Care and Use Committee of Khon Kaen University approved all of the procedures involving live animals (Record No. IACC-KKU-49/62). This experiment was conducted between April 2021 and June 2021 at the Khon Kaen University farm in Thailand. The farm’s geographical coordinates are 16.46° N latitude and 102.82° E longitude, and it is 169 m above sea level. During the experimental period, the average temperature was 36 °C, and the humidity was 78%.

In their final fattening phase, we utilized fifteen crossbred steers in the energy balance trial. These steers were Holstein Friesian Thai native crossbreeds with an 89% *Bos taurus* and 11% *Bos indicus* genetic makeup. On average, the steers were 2.5 ± 0.1 years old and had an initial body weight of 603.7 ± 14.3 kg. Before the 90-day feeding trial, the experimental animals underwent a 14-day adaptation period. We ensured their health and well-being by treating them for intestinal and external parasites (1 mL/50 kg body weight; Ivermectin F, Bangkok, Thailand) and injecting them with vitamins A, D3, and E (10 mL/head; Vitamin A propionate, 300,000 IU; Vitamin D_3_ Cholecalciferol, 100,000 IU; Vitamin E acetate, 50 mg; Phenix, Bangkok, Thailand). Throughout the experiment, the steers were housed in individual pens (2.5 m × 4.5 m dimensions) with free access to feed and drinking water and were offered food twice daily ad libitum at 08:00 a.m. and 15:00 p.m.

The experimental design used in this study, the randomized complete block design, considered the individual animals in each pen as experimental units. Three dietary treatments required fifteen animals to be assigned to one of five blocks according to the initial BW grouping. Each block randomly allotted the animals to one of three dietary treatments. The three dietary treatments in our experiment involved substituting cassava pulp with cassava chips and broken rice in the diets. These substitutions were made on dry matter basis ratios of 50:0:0, 30:20:0, or 10:20:20. The ingredients and chemical compositions of the diets are shown in Table 1. The experimental diet was formulated to meet the 1.0 kg average daily gain for the nutrient requirements of fattened beef cattle according to the guidelines of the Working Committee of Thai Feeding Standards for Ruminants (WTSRs) [17] to produce a total mixed ration using the ingredients shown in Table 2. The total mixed ration (TMR) was prepared using a vertical mixer with a 2000 kg capacity (Celikel TMR feed mixer, 108 Agriculture Machine and Equipment Co., Ltd., Lop Buri, Thailand). Around 35 kg of fermented TMR in the silo bags was ensiled and kept in an outdoor ambient temperature for no less than seven days before being offered [8].

### 2.2. Feed Intake, Digestibility, and Behaviors

Samples were collected from each animal daily during the five-day digestion trials (from 8:00 a.m. to 7:30 a.m. on the following day) [11,12]. The animals in each block were maintained in individual pens for at least 18 days before being moved in block sequence to a metabolism cage for 5 consecutive days for feces and urine collection. We recorded the daily weights of the feed offered and refused for each animal. We also collected feed samples (roughly 1 kg), which were dried at 60 °C for 72 h, ground through a 1 mm screen, and then stored for chemical composition and gross energy (GE) content analyses. To measure daily urine volume, we immediately recorded and collected samples in 5 L plastic buckets containing 6 N HCl to maintain a pH below 3.0. We then mixed the urine collected from each animal and sampled about 500 mL for the further analysis of the nitrogen and GE contents. The dry matter feed intake was calculated by subtracting the amount of feed refused from the provided amount. To calculate the apparent total tract nutrient digestibility, we compared the daily nutrient intake with the amount of the corresponding nutrient that appeared in the feces.

The cattle’s daily eating, ruminating, and other activities were monitored using an automatic data recording device, SenseHub (Allflex Livestock Intelligence, SCR Engineers Ltd., Netanya, Israel) [18].

### 2.3. Animal Calorimetry and Enteric Methane Measurements

The respiratory gas exchange measurements were conducted using the animal calorimetry system during the final three days of the digestion trials. To achieve this, we followed the methods outlined by Suzuki et al. [19], which involved utilizing four metabolic crates (with dimensions of 105 cm × 80 cm × 173 cm) equipped with a ventilated head box type and flow meter (NFHY-R-O-U, Nippon Flow Cell, Tokyo, Japan). This allowed us to measure the consumption of oxygen (O_2_) and the production of carbon dioxide (CO_2_) and methane (CH_4_) while recording the tube air flow rate (mean of 644.8 ± 2.5 L/min, standard error of the mean) and the total volume of air that flowed through the system. We analyzed the inflow and outflow tube gasses for the O_2_, CO_2_, and CH_4_ concentrations using a gas analyzer (MultiExact 4100 Analyzer, Servomex Group, East Sussex, UK). To calibrate the system, we used certified gasses from Linde (Thailand) Public Co. Ltd., Samutprakarn, Thailand, which included two O_2_ concentrations (18.90% and 24.96%), 1.79% of CO_2_, and 1760 ppm of CH_4_ daily before morning feeding. The gas recovery test was conducted using the CO_2_ injection method, which gave us recovery values ranging from 98% to 104%. We measured gas data at 7.5 min intervals for 23.30 h daily (from 8:00 a.m. to 7:30 a.m. on the following day) during the last three days of the digestion trial. These measurements were adjusted to 24 h to determine the total respiratory gas exchange.

The energy partitioning method was conducted according to Gunha et al. [11,12], and it included the gross energy intake, the energy from feces and urine, methane, heat production, and the energy balance. We determined the metabolizable energy content by subtracting the energy outputs from urine and methane from the digestible energy. Digestible energy determination was achieved by subtracting energy outputs from the fecal and gross energy intakes [11,12]. To calculate daily heat production, Brouwer’s equation [20] was applied, which is expressed as heat production (kJ/d) = (16.18 × O_2_) + (5.02 × CO_2_) − (2.17 × CH_4_) − (5.99 × N). In this equation, O_2_, CO_2_, and CH_4_ represent the volumes of oxygen, carbon dioxide, and methane produced (L/day), while N is the quantity of urinary nitrogen excreted (g/day). The researchers converted CH_4_ production into energy using a conversion factor of 39.54 kJ/L.

### 2.4. Chemical Analyses

The samples of the offered feed, refused feed, feces, and urine were analyzed to determine their chemical compositions. The dry matter content was measured by oven-drying at 105 °C until a constant weight was reached. The subsamples were then dried in an oven at 60 °C, milled, passed through a 1 mm screen, and subjected to a chemical analysis. Ash and ether extract (EE) were determined using the AOAC methods [21]. Total nitrogen was analyzed using the DUMAS method (LECO FP828, MI, USA) and converted to crude protein (CP) using a factor of 6.25 [22]. Neutral detergent fiber (NDF) was analyzed using a fiber analyzer (ANKOM 200/220, ANKOM Technology, Macedon, NY, USA) with ash-corrected α-amylase treated with the addition of sodium sulfite, acid detergent fiber (ADF), and acid detergent lignin (ADL) [23,24]. The gross energy contents of the samples were determined using a bomb calorimeter (IKA C2000 Basic, Staufen, Germany).

The fermentation profiles and microbial populations were determined using three samples per dietary treatment. Approximately 1 kg of the silage samples was taken from each bag silo, homogeneously mixed, and divided into three subsamples. The first subsamples were chosen for chemical analysis. About 20 g of the second subsample was homogenized with 180 mL of sterilized distilled water and stored overnight at 4 °C. The sample was then filtered through filter paper (Whatman Grade No. 4 Filter Paper; Buckinghamshire, UK), and the acidity of the filtrate was immediately determined using an electrode pH meter (pH 700; Eutech Instruments, Pte Ltd., Ayer Rajah Crescent, Singapore) and then centrifuged at 9600× *g* for 10 min (LC-200 Flexpin, Tomy Digital Biology, Japan). The supernatant fluids were stored at −20 °C until further analyses. The supernatants were analyzed for ammonia nitrogen (NH_3_-N), lactic acid, and volatile fatty acid (VFA). The concentration of NH_3_-N was determined using the Kjeldahl nitrogen analyzer (VAPODEST 200, Gerhardt, Königswinter, Germany) according to the AOAC method 984.13. The lactic acid and VFA concentrations were determined using a gas chromatograph (GC2014, Shimadzu, Kyoto, Japan) equipped with a flame ionization detector and a 25 m × 0.53 mm capillary column (BPX5, SGE Analytical Science, Victoria, Australia).

The analysis of the morphological characteristics of the ensilaged bacteria, yeast, and mold involved enumeration using the agar plate count method and differentiation based on the colony and cell morphologies. The microbiological results are reported as colony-forming units (cfu) per gram of dry matter (DM), with all the data being transformed to the logarithmic (log10) scale following the method used by Kotupan and Sommart [10].

Blood and rumen fluid samples were taken from each animal three hours after morning feeding (11:00 a.m.) on day 85 of the experiment. A blood sample was also collected from each animal. Approximately 10 mL was taken from the jugular vein using a sterilized vacuum tube (Greiner Bio-One (Thailand) Ltd., Chonburi, Thailand), packed on ice, and transported to the laboratory within 1 h for plasma analyses. Colorimetric method test kits (Roche Diagnostics, Indianapolis, IN, USA) and an automated analyzer (COBAS INTEGRA 400 plus analyzer, Roche Diagnostics, Indianapolis, IN, USA) were used to determine the plasma metabolite concentrations (urea nitrogen, glucose, triglyceride, cholesterol, total protein, and albumin). Using an esophageal–rumen stomach tube technique, 200 milliliters of rumen fluid was collected from each animal. The ruminal fluid pH was measured immediately with a glass electrode pH meter (Eutech pH 700, Eutech Instruments Pte Ltd., Ayer Rajah Crescent, Singapore). Four layers of gauze were used to separate the ruminal fluids from the feed particles, and then 100 mL of rumen fluid was placed into 150 mL plastic containers with 10 mL of 6 N HCL. They were then collected and stored in ice buckets before being transported to the laboratory and stored at −20 °C. The concentration of NH_3_-N was analyzed using a Kjeldahl nitrogen analyzer. The volatile fatty acid concentrations were analyzed according to the method described by Porter and Murray [24] by using a gas chromatograph (GC2014, Shimadzu, Tokyo, Japan) equipped with a flame ionization detector and a 25 m × 0.53 mm capillary column (BPX5, SGE Analytical Science, Victoria, Australia).

### 2.5. Statistical Analysis

The data were submitted to an analysis of variance (ANOVA) using the generalized linear model procedure of SAS version 9.0 (SAS Institute Inc., Cary, NC, USA) [25]. The analysis followed a randomized complete block design, with the model including terms for the treatment (df = 2) and block (df = 4) as per the following model:

Yij = μ + τi + βj + εij

Here, Yij represents the dependent variable, μ denotes the overall mean, τi refers to the fixed effect of dietary treatment (i = from 1 to 3), βj represents the fixed effect of the block (j = from 1 to 5), and εij denotes the residual error. Duncan’s new multiple-range test was employed with a significance level set at *p* < 0.05 to determine the statistical difference between the treatment means.

## 3. Results

### 3.1. Dietary Treatments

Table 1 and Table 2 show the feed ingredients and formula used to analyze the chemical compositions of the diets. Table 3 shows the dietary treatment’s ensiling fermentation profiles and microbial populations. Silage pH and acetic acid concentration reductions (*p* < 0.01) occurred when more cassava pulp was added to the diets. There was no significant difference (*p* > 0.05) in the lactic acid, acetic acid, propionic acid, butyric acid, and ammonia nitrogen concentrations. The lactic acid bacteria counts were increased (*p* < 0.05), whereas those for aerobic bacteria, yeasts, and molds did not differ (*p* > 0.05) among the dietary treatments. Coliform bacteria were not detected in the silage fermented within seven days.

### 3.2. Feed Intake and Apparent Total Tract Nutrient Digestion

When cassava pulp was substituted for broken rice (Table 4), there was no significant difference (*p* > 0.05) among the dietary treatments in the digestibility of dry matter, organic matter, crude protein, ether extract, and fiber, but the feed intake was increased (*p* < 0.01) significantly. The daily time spent eating, ruminating, and performing activities did not significantly differ (Table 4).

### 3.3. Ruminal Fermentation Characteristics and Blood Metabolites

The dietary treatment did not affect the ruminal pH and total VFA, propionate, acetate, iso-butyrate, iso-valerate, or valerate concentrations (Table 5). In contrast, the ammonia nitrogen and butyrate concentrations increased (*p* < 0.05), while the acetate/propionate ratio decreased (*p* < 0.05) when substituting cassava pulp with broken rice and cassava chips. The blood metabolites were not affected by the diets (Table 5).

### 3.4. Respiratory Gas Exchange and Enteric Methane Emissions

**Figure 1:**
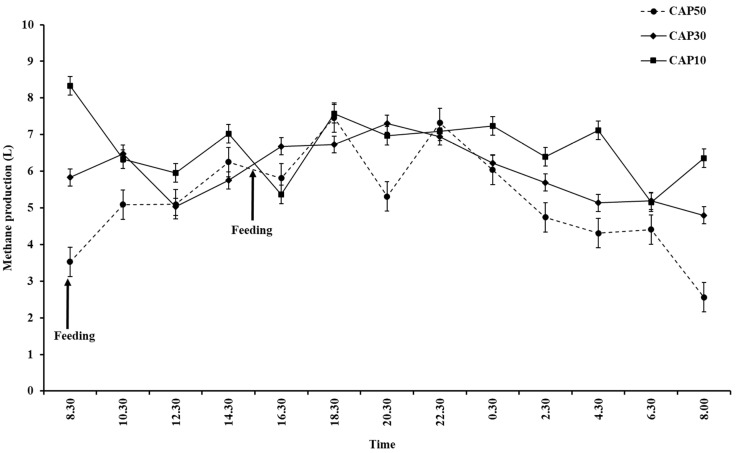
Mean and standard error (bars) of two hourly enteric methane emission patterns measured in respiration chamber from Holstein crossbred cattle that underwent dietary treatments. Symbols are data from five cattle that underwent diet treatments for three consecutive days: CAP50 = 50% cassava pulp + 0% cassava chip + 0% broken rice (●); CAP30 = 30% cassava pulp + 20% cassava chip + 0% broken rice (◆); CAP10 = 10% cassava pulp + 20% cassava chip + 20% broken rice (■). Feeding times: morning (8:30 h) and afternoon (15:00 h).

Table 6 shows the respiratory gas exchange and enteric methane emission data. The dietary treatments did not affect (*p* > 0.05) oxygen consumption, carbon dioxide production, or methane production, yield, intensity, and conversion rates. The enteric methane emission pattern showed a quadratic curve response to morning and afternoon feeding (Figure 1). The time taken to reach peak methane emission was three hours after feeding, and then that of all animals that were fed the diets gradually declined until the following day of feeding.

### 3.5. Energy and Nitrogen Partition

The dietary treatments did not affect (*p >* 0.05) energy loss in the feces, methane emission, or heat production; however, the gross energy intake, energy loss in the urine, energy balance, nitrogen intake, and nitrogen balance were increased (*p* < 0.05) when substituting cassava pulp with broken rice and cassava (Table 7). When more cassava pulp was added to the diet, the intake of gross, digestible, and metabolizable energy (*p* < 0.01) was significantly reduced.

## 4. Discussion

### 4.1. Chemical Compositions of Diets

The chemical composition of broken rice indicated that it has superior nutrient density and availability and, thus, a greater energy supply than cassava pulp and cassava chips. However, it is important to note that the cost of broken rice diets is significantly higher than those of cassava chip and pulp diets. These findings have practical implications for low-cost feeding and the development of nutritionally balanced ruminant diets. Because the final phase feeding of fattened beef requires a high energy intake, feed calculation requires a high level of grain with an optimum roughage ratio in the diets [2,10,16]. The commonly recommended proportion of rice grain in the diets of fattened beef cattle ranges from 20% to 36% [13,14,15,16]. Thailand produces 32 million tons of rice annually and approximately 4.48 million tons of broken rice, which is a by-product of rice milling used as feed. However, the seasonal production and overproduction of rice in some years has lowered the world’s rice export prices. Broken rice can be used as an alternative feed resource for corn grain or cassava chips, especially when the price of imported corn grain is high.

In this study, the nutritional value of the diet, particularly the protein and fat contents, increased when cassava pulp was replaced with broken rice and cassava chips. The relatively higher crude protein (15.0 vs. 14.3 vs. 14.0%) content could be attributed to the protein content in broken rice compared with that in cassava chips and cassava pulp. Additionally, the greater NDF, ADF, and ADL contents and lower NFC contents of cassava pulp were found in the more fibrous diets when cassava pulp was substituted with broken rice and cassava chips. These differences in the fiber content suggest that the lignocellulose content in cassava pulp is higher than that of broken rice and cassava chips. These results confirm that replacing cassava pulp with concentrate in cattle diets increases the lignocellulose content [9]. The dietary fiber content in this study ranged from 29.4 to 43.2%, which falls within the recommended range for stimulating rumination and maintaining rumen health [26].

### 4.2. Ensiling Fermentation Profiles and Microbial Populations

Silage contains lactic acid and volatile fatty acids, such as acetic, propionic, and butyric acids, as the fermentation end-products of ensiling microbiota [8,10,27]. Under normal feeding conditions, lactic acid from silage is converted to propionate in the rumen, where it can be absorbed for glucose synthesis via gluconeogenesis and then used as a substrate for fatty acid incorporated into the milk or body fat of the host ruminant [9,10,11,28]. One of the most significant results was that the silage pH was increased by up to 30% when cassava pulp was substituted with broken rice or cassava chips in the diets. This study’s higher basicity (pH range from 3.7 to 3.9) is strongly associated with the lower lactic acid bacteria count (Table 2). During ensiling, lactic acid produced by bacteria is usually found in the highest concentration in silage. Its strong acidity contributes the most to the decline in silage pH during ensiling fermentation [10,11,12]. It plays a vital role in stabilizing silage fermentation by inhibiting the growth of microbiota that are intolerant to a low pH, such as coliform bacteria and yeast [27]. The recommended typical high-moisture corn silage pH should range from 4.0 to 4.5, with a high lactic acid (from 0.5 to 2.0% of dry matter basis) content and trace amounts of acetic acid and ammonia [27]. In our previous work, we demonstrated that a total mixed ration silage prepared by mixing wet by-products, such as cassava pulp and brewer’s grains, with dry feed ingredients, such as rice straw and oil cake, and preserving them as silage produced an excellent fermentation profile that was well preserved, demonstrating a pH lower than <4.2 within 7 d of ensilage [2,7,10,27]. This work highlights the advantages and greater availability of water-soluble carbohydrates for lactic acid bacteria growth in cassava pulp than in cassava chips or broken rice, suggesting that high-quality silage was produced. This finding can potentially contribute to the development of silage feed and feeding technology based on cassava pulp agro-industrial by-products in the tropics.

### 4.3. Ruminal Fermentation Characteristics and Blood Metabolites

The results of this study indicate that the rumen pH was similar among the dietary treatments, ranging from 6.54 to 6.76, which is within the normal range of 6.0 to 7.0 [26]. The rumen pH is critical to the microbiota fermentation process and rumen health [26]. The pH for rumen microorganisms is responsible for digestion, and fermentation turns the components into short-chain volatile fatty acid, ammonia nitrogen, and microbial biomass [2,10]. This study’s results agree with those of another report showing no sign of rumen acidosis illness, including reduced feed intake, diarrhea, laminitis, and body weight loss [2,10,11,12]. These data indicate that the animals were in good rumen health as the diets’ NDF content (range from 29.4 to 43.2%) is not only sufficient for Holstein cattle’s fiber requirement, but also crucial for stimulating ruminating activity and maintaining a ruminal pH that is greater than 5.6 to avoid sub-acute rumen acidosis [2,26].

Because the final phase feeding of fattened beef requires a high energy intake, a high amount of grain and an optimum dietary forage fiber or roughage ratio are required in the diets. In this study, the dietary NDF ranged from 29.4% to 43.2% (see Table 2), resulting in the daily rumination time ranging from 6.96 to 7.58 h and the ruminal pH ranging from 6.54 to 6.76, indicating that the diets provided sufficient dietary NDF for rumen health [26]. Our previous work indicates that 10% of the rice straw included in the total mixed ration of native Thai cattle [8] and Holstein crossbred bulls [2] maintained an average daily 8.5-to-9.85 h chewing time and 3.1-to-4.7 h ruminating time, suggesting that most tropical feed and systems depend on agricultural industry by-products that have a high NDF and effective NDF fraction, such as rice straw, cassava pulp, and palm kernel cake. This contributes to rumen health and stimulates sufficient ruminating activities (from 2.5 to 10.5 h/d) [2,11,12]. This study’s estimated total forage NDF concentration ranges from 15.9% to 24.1%, suggesting that the diets provided sufficient dietary NDF [26]. The NRC [26] recommended the minimum concentration of total dietary NDF of 25% with the condition that 19% of the dietary NDF is from forage. In this study, rice straw is the main forage NDF, with the non-forage source contributions from cassava pulp and palm kernel cakes ranging from 29.4% to 43.2% (see Table 2); therefore, the physical structure of rice straw forage and non-forage fiber sources may be expected to remain sufficient for chewing and ruminating, thereby promoting good rumen function and cattle health [2,11].

Rumen ammonia nitrogen primarily comes from microorganisms’ degradation of dietary protein [26]. This is critical in rumen fiber digestion and microbial protein synthesis [26,29]. In this study, the amount of ammonia increased substantially when broken rice was included in the diet, indicating a higher loss of protein that is broken down extensively during fermentation by rumen microbial enzymes. More protein was lost than that in the cassava pulp diets. The reason may be that broken rice has a higher quantity of rumen-degraded protein, which is attributed to the higher protein content in broken rice compared with those in the cassava chip and cassava pulp diets. In this study, the ammonia concentration did not differ between the broken rice and cassava chip diets. Kotupan and Sommart [10] observed no difference between cassava and broken rice in the fermented total mixed ration-fed fattened beef cattle. Also, Yoo et al. [16] reported no difference between corn and rice in the total mixed ration of fattened Hanwoo beef cattle. The similar ruminal ammonia concentration suggests that broken rice and cassava diets have a similar rumen-undegradable protein value. Moreover, the ammonia concentration in this study ranged between 3.61 and 6.12 mg/dL, indicating adequate levels for efficient rumen fermentation and microbial protein synthesis, as reported in the literature [29].

Regarding volatile fatty acid, the rumen fermentation process did not affect the total quantity of volatile fatty acid. However, the short-chain acetate/propionate fatty acid ratio increased in the broken rice diet, suggesting that the rumen microbiota and fermentation end-products shifted during the degradation of the NFC fraction [10,14]. These results align with the previously reported findings indicating that replacing cassava chips with broken rice in fattened beef cattle diets increases the quantity of propionate [10]. In this study, substituting cassava pulp with cassava chips and broken rice suggests an increased propionate, while the acetate content and A:P ratio decreased with the increasing energy density in the diets. Rumen bacteria can use energy-dense grains to produce propionate, a precursor for glucose synthesis by gluconeogenesis; thus, glucose is further used as a precursor for fatty acid biosynthesis in ruminants [2,10,28]. Similarly, it has been reported that as the percentage of propionate increased, the decrease in the acetate-to-propionate ratio was influenced by the type of dietary degradable carbohydrate; the cassava chip-rich diet and broken rice-rich diet were substituted for cassava pulp [10,11,12]. The proportion of butyrate was no different in the broken rice-rich diet than it was in the cassava chip-rich diet. This is similar to the findings of Kotupan and Sommart [10], who showed no difference when replacing cassava chips with broken rice in fattened beef cattle diets. However, the proportion of butyrate was higher in the broken rice diets than it was in the cassava pulp diets. These results indicate alterations in the diet composition and microbial population in the rumen.

Enteric methane is a greenhouse gas generated by rumen methanogens that capture hydrogen and carbon dioxide as energy sources, indicating methane energy loss in the rumen fermentation process [2,30]. In the present study, the diets did not significantly change the daily methane emissions, suggesting a smaller greenhouse gas footprint for cassava pulp, which is not edible for humans, than those for cassava chip and broken rice. These confirm a similar feed intake, digestibility, and rumen fermentation among the dietary treatments. Still, there is limited research that involves monitoring the enteric methane produced when cassava pulp is used as a high-energy ruminant feed source compared to broken rice or cassava chips. Authors such as Molona-Botero et al. [5] recently reported no difference among diets supplemented with cassava root, with 7.2% to 9.3% methane conversion rate values being found in tropical dairy cows fed cassava chip-based diets. Binsulong et al. [2] indicate that replacing rice straw with cassava pulp at a concentration ranging from 5% to 35% in Holstein bulls’ rations substantially reduced their enteric methane emissions. These results suggest that cassava pulp can be used as an alternative feed to enhance nutrient and energy supplies without causing an environmental impact on ruminants.

National greenhouse gas inventories use the methane conversion factor (Ym) to assess the impact of global warming potential [30]. In this study, the methane conversion factor ranged from 6.9 to 7.9%, indicating a higher value than the IPCC’s default value of 6.3% for cattle and buffalo fed >75% grain and silage [30]. Also, Gunha et al. [12] reported 6.4% to 7.7% methane conversion rate values in tropical dairy cows fed cassava chip-based diets. These results are consistent with previous works [2,8] reporting a high enteric methane conversion factor of zebu cattle fed low-quality roughage-based diets. The typically high lignocellulose content of feed resources in tropical cattle production systems may be the main factor affecting methane emissions. Improving the feed quality can be an essential future strategy to reduce enteric methane emissions.

### 4.4. Feed Intake, Nutrient Digestion, and Energy Balance

Feed intake and digestibility are significant factors that control the energy supply required to maintain and produce fattened beef cattle [2]. These energy requirements can be achieved by providing highly digestible energy feed sources and increasing metabolizable energy to enhance the animals’ productivity [11,12]. This study found no differences in organic matter digestibility or methane energy loss but a significant feed intake, indicating a greater metabolizable energy supply for maintenance and production when adding cassava chips and broken rice to the diets. Although the fiber content was higher when cassava pulp was substituted in the diets, digestibility may not be affected because cassava pulp contains a higher hemicellulose fraction that is readily digestible fiber in the rumen. Still, limited research compares cassava pulp with grains as an energy feed source in ruminants. Moreover, these results also agree with previous works [15] reporting that using rice grain and not corn grain in the diets of fattened beef cattle improves the digestibility of starch and protein, ruminal propionate, and the marbling score. Botero et al. [5] also reported no differences in the dry and organic matter intakes when the cows ate cassava roots. Kotupan and Sommart [10] showed no difference in nutrient intake and digestibility when replacing cassava chips with broken rice in fattened beef cattle diets. Yoo et al. [16] reported that when rice grain was substituted with corn grain, it had no adverse effects on the growth performance, rumen fermentation characteristics, and blood metabolites of Hanwoo steers.

These results suggest using strategic feeding with broken rice to enhance nutrients and energy supplies. We recommend further research on beef cattle nutrition and engagement to provide a more comprehensive understanding of the practical implications for farmers, animal nutritionists, veterinarians, and technicians in feeding and management [31].

However, this study’s limitations include conducting short-term metabolism trials and the number of animals studied. Fattened beef production performance may need to be confirmed in a long-term feeding experiment.

## 5. Conclusions

Broken rice offers superior nutrient and energy availability compared to cassava pulp and chips. However, cassava pulp and chips positively influence the ensilage pH, the lactic acid bacteria count, and the ruminal fermentation characteristics without adversely affecting enteric methane emissions. These findings highlight the potential of broken rice and suggest the possibility of strategic feeding strategies that could enhance nutrient and energy supplies while minimizing environmental impacts.

## Figures and Tables

**Table 1:** An analysis of the chemical compositions of broken rice, cassava pulp, and cassava chips.

Item	Feedstuffs		
	Cassava Pulp	Cassava Chip	Broken Rice
Chemical composition, % of DM			
Dry matter (DM)	19.5	88.8	88.9
Organic matter	96.8	96.7	99.2
Crude protein	2.7	2.5	7.4
Ether extract	0.4	0.8	1.3
Non-fiber carbohydrates	74.5	89.7	84.9
Acid detergent fiber	25.1	5.3	1.2
Acid detergent lignin	2.5	0.4	0.1
Neutral detergent fiber	38.9	13.7	6.4
Feed cost, THB/kg DM ^1^	2.82	7.77	13.08

^1^ The current exchange rate is THB 36.8 = USD 1.

**Table 2:** Ingredients, feed costs, analyzed chemical compositions, and energy contents of dietary treatments.

	Treatment ^1^		
Item	CAP50	CAP30	CAP10
Ingredients (% DM)			
Rice straw	10	10	10
Cassava pulp	50	30	10
Cassava chip	-	20	20
Broken rice	-	-	20
Wet brewery	10	10	10
Palm meal	11	11	11
Rice bran	10	10	10
Soybean meal	7.5	7.5	7.5
Urea	0.5	0.5	0.5
Mineral ^2^	0.9	0.9	0.9
Premixed ^3^	0.1	0.1	0.1
Total	100	100	100
Chemical composition (% DM)			
Dry matter	34.7	36.4	36.4
Organic matter	93.5	93.5	93.6
Crude protein	14.0	14.3	15.0
Ether extract	3.74	4.18	4.98
Neutral detergent fiber	43.2	40.8	29.4
Acid detergent lignin	3.65	3.51	3.26
Acid detergent fiber	24.3	20.4	15.7
Non-fiber carbohydrate ^4^	32.6	34.2	44.0
Energy content (MJ/kg DM) ^5^			
Gross energy	18.1	18.1	18.3
Digestible energy	12.6	13.3	13.6
Metabolizable energy	10.5	11.3	11.8

^1^ CAP50 = 50% cassava pulp + 0% cassava chip + 0% broken rice; CAP30 = 30% cassava pulp + 20% cassava chip + 0% broken rice; CAP10 = 10% cassava pulp + 20% cassava chip + 20% broken rice. ^2^ Minerals included 93.72 g Ca, 46.86 g P, 107.78 g Na, 18.55 g S, 8.24 g Mn, 7.49 g Zn, 3.37 g Mg, 1.17 g Cu, 0.15 g Co, 0.01 g K, 0.04 g I, and 0.02 g Se. ^3^ Premix included 5,000,000 IU vitamin A, 1,000,000 IU vitamin D_3_, 10,000 IU vitamin E, 25 g Fe, 4 g Cu, 20 g Mg, 0.13 g Co, 15 g Zn, 0.75 g I, 0.38 g Se, and 0.88 g feed additive. ^4^ Calculated according to the following formula: 1000 − (crude protein + neutral detergent fiber + ether extract + ash). ^5^ Determined energy content using animal calorimetry method, each from five Holstein crossbred cattle that underwent dietary treatments measured in respiration chamber for three consecutive days [10].

**Table 3:** Ensiling fermentation profiles and microbial populations of dietary treatment.

Item	Treatment ^1^			SEM	*p*-Value
	CAP50	CAP30	CAP10		
Ensiling fermentation profile					
pH	3.70 ^b^	3.76 ^b^	3.89 ^a^	0.03	0.01
Lactic acid (g/kg DM)	23.7	16.7	31.4	5.56	0.25
Acetic acid (g/kg DM)	10.6	8.03	14.4	2.02	0.16
Propionic acid (g/kg DM)	1.29	0.96	2.00	0.36	0.20
Butyric acid (g/kg DM)	0.31	0.21	0.26	0.04	0.31
NH_3_-N (g/kg total N)	19.0	20.0	21.0	2.94	0.89
Microbial populations (log_10_ cfu/g DM)					
Lactic acid bacteria	5.72 ^ab^	6.19 ^a^	4.71 ^b^	0.34	0.05
Coliform bacteria	ND	ND	ND	-	-
Aerobic bacteria	7.62	7.89	7.44	0.36	0.69
Yeasts	4.21	5.65	6.26	1.14	0.28
Molds	6.66	6.32	5.85	0.32	0.47

^1^ CAP50 = 50% cassava pulp + 0% cassava chip + 0% broken rice; CAP30 = 30% cassava pulp + 20% cassava chip + 0% broken rice; CAP10 = 10% cassava pulp + 20% cassava chip + 20% broken rice. ^a,b^ Mean values in same row with different superscripts differ significantly (*p* < 0.05). ND = not detected.

**Table 4:** Feed intake, apparent total tract nutrient digestion, and behaviors of fattening crossbred Holstein Friesian cattle that underwent dietary treatments.

Item	Treatment ^1^			SEM	*p*-Value
	CAP50	CAP30	CAP10		
Feed intake (kg DM/d)	4.73 ^b^	6.35 ^a^	7.58 ^a^	0.30	0.01
Apparent nutrient digestion (%)					
Dry matter	73.9	71.4	73.5	0.80	0.37
Organic matter	72.1	75.4	80.2	2.75	0.14
Crude protein	68.2	71.2	77.2	2.06	0.06
Ether extract	80.6	84.7	90.7	3.16	0.09
Neutral detergent fiber	62.8	62.0	51.8	5.02	0.82
Acid detergent fiber	43.8	40.8	39.1	4.28	0.97
Non-fiber carbohydrate	87.9	87.1	67.5	16.7	0.77
Behavior (h/d)					
Eating activity	1.78	1.65	0.94	0.23	0.85
Ruminating activity	7.32	6.96	7.58	0.72	0.78
Others activity ^2^	2.54	4.01	3.21	0.40	0.10

Abbreviations: DM, dry matter; SEM, standard error of means. ^1^ CAP50 = 50% cassava pulp + 0% cassava chip + 0% broken rice; CAP30 = 30% cassava pulp + 20% cassava chip + 0% broken rice; CAP10 = 10% cassava pulp + 20% cassava chip + 20% broken rice. ^2^ Walking and standing. ^a,b^ Mean values in the same row with different superscripts differ significantly (*p* < 0.05).

**Table 5:** Ruminal fermentation characteristics and blood biochemical metabolites of fattening crossbred Holstein Friesian cattle that underwent dietary treatments.

		Treatment ^1^			
Item	CAP50	CAP30	CAP10	SEM	*p*-Value
Ruminal fermentation					
pH	6.76	6.54	6.66	0.10	0.37
NH_3_-N (mg/dL)	3.61 ^b^	4.46 ^ab^	6.12 ^a^	0.57	0.04
Total VFA (mmol/L)	103	100	86.1	6.43	0.26
Acetic acid (%)	72.2	70.1	64.3	2.23	0.09
Propionic acid (%)	15.2	14.7	19.6	1.22	0.06
Iso-butyric acid (%)	1.06	1.01	0.86	0.23	0.74
Butyric acid (%)	7.91 ^b^	10.8 ^a^	11.1 ^a^	0.59	0.02
Iso-valeric acid (%)	2.41	2.71	2.70	0.66	0.88
Valeric acid (%)	1.19	0.62	1.28	0.21	0.40
Acetic: propionic acid ratio	4.83 ^a^	4.77 ^a^	3.46 ^b^	0.33	0.04
Blood biochemical metabolites					
Urea-N (mg/dL)	10.2	10.8	11.0	1.29	0.90
Glucose (mg/dL)	69.8	69.6	71.6	0.96	0.32
Triglyceride (mg/dL)	82.8	86.2	92.0	5.52	0.52
Cholesterol (mg/dL)	20.8	24.0	22.6	2.56	0.69
Total protein (g/dL)	7.14	6.98	7.12	0.16	0.76
Albumin (g/dL)	3.70	3.88	3.70	0.07	0.20

Abbreviations: NH_3_-N, ammonia nitrogen; VFA, volatile fatty acid; SEM, standard error of means. ^1^ CAP50 = 50% cassava pulp + 0% cassava chip + 0% broken rice; CAP30 = 30% cassava pulp + 20% cassava chip + 0% broken rice; CAP10 = 10% cassava pulp + 20% cassava chip + 20% broken rice. ^a,b^ Mean values in the same row with different superscripts differ significantly (*p* < 0.05).

**Table 6:** Respiratory gas exchange and enteric methane emissions of crossbred Holstein Friesian fattening cattle that underwent dietary treatments.

		Treatment ^1^			
Item	CAP50	CAP30	CAP10	SEM	*p*-Value
Respiratory gas exchange					
Oxygen consumption (L/d)	2817	3022	3027	182	0.44
Carbon dioxide production (L/d)	3079	3577	3369	216	0.36
Respiratory quotient	1.09	1.20	1.11	0.02	0.50
Enteric methane emissions					
Methane production (L/d)	181	226	227	22.5	0.28
Methane production (g/d)	129	163	162	16.1	0.28
Methane yield (g/kg DMI)	22.9	25.9	24.8	1.50	0.38
Methane intensity (g/kg of weight gain)	6.72	5.14	6.06	1.25	0.81
Methane energy (MJ/d)	7.15	8.99	8.94	0.89	0.28
Methane conversion factor (%)	6.97	7.91	7.50	0.54	0.47

Abbreviations: DMI, dry matter intake; SEM, standard error of means; respiratory quotient = CO_2_ production/O_2_ consumption; GEI, gross energy intake; methane conversion factor = methane energy × 100/GEI (%). ^1^ CAP50 = 50% cassava pulp + 0% cassava chip + 0% broken rice; CAP30 = 30% cassava pulp + 20% cassava chip + 0% broken rice; CAP10 = 10% cassava pulp + 20% cassava chip + 20% broken rice.

**Table 7:** Energy intake, energy partition, and nitrogen partition of crossbred Holstein Friesian fattening cattle that underwent dietary treatments.

		Treatment ^1^			
Item	CAP50	CAP30	CAP10	SEM	*p*-Value
Energy intake					
Gross energy (MJ/d)	85.5 ^b^	115 ^a^	138 ^a^	5.71	<0.01
Digestible energy (MJ/d)	59.9 ^b^	84.7 ^a^	103 ^a^	4.49	<0.01
Metabolizable energy (MJ/d)	49.6 ^b^	72.2 ^a^	88.9 ^a^	4.36	0.01
Energy partition (kJ/kg BW^0.75^)					
Gross energy intake	674 ^c^	921 ^b^	1111 ^a^	39.73	<0.01
Fecal excretion	203	242	290	43.8	0.36
Urine excretion	24.7 ^b^	28.3 ^b^	38.7 ^a^	2.82	0.04
Methane emission	56.7	72.4	72.7	6.60	0.20
Heat production	463	519	504	23.2	0.22
Energy balance	−73.3 ^c^	59.7 ^b^	206 ^a^	29.73	0.01
Nitrogen (N) partition, g/d					
Intake N	106 ^b^	145 ^ab^	183 ^a^	11.6	0.02
Fecal N	35.7	42.6	53.3	12.5	0.50
Urine N	59.9	61.2	84.3	13.2	0.27
N balance	10.4 ^b^	41.0 ^ab^	45.0 ^a^	9.26	0.05

Abbreviations: DM, dry matter; BW^0.75^, metabolic body weight; SEM, standard error of means: RQ, respiratory quotient = CO_2_ production/O_2_ consumption. ^1^ CAP50 = 50% cassava pulp + 0% cassava chip + 0% broken rice; CAP30 = 30% cassava pulp + 20% cassava chip + 0% broken rice; CAP10 = 10% cassava pulp + 20% cassava chip + 20% broken rice. ^a,b,c^ Mean values in the same row with different superscripts differ significantly (*p* < 0.05).

## Data Availability

The data are available upon reasonable request to the corresponding author.

## References

[1] Masebo N.T., Marliani G., Cavallini D., Accorsi P.A., Di Pietro M., Beltrame A., Gentile A., Jacinto J.G.P. (2023). Health and welfare assessment of beef cattle during the adaptation period in a specialized commercial fattening unit. Res. Vet. Sci..

[2] Binsulong B., Gunha T., Kongphitee K., Maeda K., Sommart K. (2023). Enteric methane emissions, rumen fermentation characteristics, and energetic efficiency of Holstein crossbred bulls fed total mixed ration silage with cassava instead of rice straw. Fermentation.

[3] Sommart K., Wanapat M., Rowlinson P., Parker D.S., Climee P., Panishying S. (2000). The use of cassava chips as an energy source for lactating dairy cows fed with rice straw. Asian-Australas. J. Anim. Sci..

[4] Wanapat M., Kang S. (2015). Cassava chip (*Manihot esculenta* Crantz) as an energy source for ruminant feeding. Anim. Nutr..

[5] Molina-Botero I.C., Gaviria-Uribe X., Rios-Betancur J.P., Medina-Campuzano M., Toro-Trujillo M., González-Quintero R., Ospina B., Arango J. (2024). Methane emission, carbon footprint and productivity of specialized dairy cows supplemented with bitter cassava (Manihot esculenta Crantz). Animals.

[6] The Thai Tapioca Trade Association Statistics of Tapioca Production in Thailand. http://ttta-tapioca.org.

[7] Jakrawatana N., Pingmuangleka P., Gheewala S.H. (2016). Material flow management and cleaner production of cassava processing for future food, feed, and fuel in Thailand. J. Clean. Prod..

[8] Kongphitee K., Sommart K., Phonbumrung T., Gunha T., Suzuki T. (2018). Feed intake, digestibility and energy partitioning in beef cattle fed diets with cassava pulp instead of rice straw. Asian-Australas. J. Anim. Sci..

[9] Zheng Y., Zhao Y., Xue S., Wang W., Wang Y., Cao Z., Yang H., Li S. (2021). Feeding value assessment of substituting cassava (Manihot esculenta) residue for concentrate of dairy cows using an In Vitro gas test. Animals.

[10] Kotupan K., Sommart K. (2021). Broken rice in a fermented total mixed ration improves carcass and marbling quality in fattened beef cattle. Anim. Biosci..

[11] Gunha T., Kongphitee K., Binsulong B., Sommart K. (2023). The Energy contents of broken rice for lactating dairy cows. Animals.

[12] Gunha T., Kongphitee K., Binsulong B., Sommart K. (2023). Net energy value of a cassava chip ration for lactation in Holstein–Friesian crossbred dairy cattle estimated by indirect calorimetry. Animals.

[13] Scheibler R.B., Schafhäuser J., Rizzo F.A., Nörnberg J.L., Vargas D.P., Silva J.L.S., Fluck A.C., Fioreze V.I. (2015). Replacement of corn grain by brown rice grain in dairy cow rations: Nutritional and productive effects. Anim. Feed Sci. Technol..

[14] Miyaji M., Haga S., Matsuyama H., Hosoda K. (2016). Effect of feeding brown rice instead of corn on lactation performance and blood metabolites in periparturient dairy cows. Anim. Feed Sci. Technol..

[15] Yang S., Kim B., Kim H., Moon J., Yoo D., Baek Y.-C., Lee S., Seo J. (2020). Replacement of corn with rice grains did not alter growth performance and rumen fermentation in growing Hanwoo steers. Asian-Australas. J. Anim. Sci..

[16] Yoo D., Yang S., Kim H., Moon J., Seo J. (2023). Effects of the use of rice grain on growth performances, blood metabolites, rumen fermentation, and rumen microbial community in fattening Hanwoo steers. Animals.

[17] Working Committee of Thai Feeding Standard for Ruminant (WTSR) (2010). Nutrient Requirement of Beef Cattle in Indochinese Peninsula.

[18] Heinrichs A.J., Heinrichs B.S., Cavallini D., Fustini M., Formigoni A. (2020). Limiting total mixed ration availability alters eating and rumination patterns of lactating dairy cows. J. Dairy Sci..

[19] Suzuki T., Phaowphaisal I., Pholsen P., Narmsilee R., Indramanee S., Nitipot P., Chaokaur A., Sommart K., Khotprom N., Panichpol V. (2008). In vivo nutritive value of Pangola grass (*Digitaria eriantha*) hay by a novel indirect calorimeter with a ventilated hood in Thailand. Japan Agric. Res. Q..

[20] Brouwer E., Blaxter K.L. (1965). Report of subcommittee on constants and factors. Energy Metabolism of Farm Animals.

[21] AOAC (1995). Official Methods of Analysis.

[22] Etheridge R.D., Pesti G.M., Foster E.H. (1998). A Comparison of nitrogen values obtained utilizing the Kjeldahl nitrogen and dumas combustion methodologies (LecoCNS2000) on samples typical of an animal nutrition analytical laboratory. Anim. Feed Sci. Technol..

[23] Van Soest P.J., Robertson J.B., Lewis B.A. (1991). Methods for dietary fiber, neutral detergent fiber, and non-starch polysaccharides in relation to animal nutrition. J. Dairy Sci..

[24] Mertens D.R. (2002). Gravimetric determination of amylase-treated neutral detergent fiber in feeds with refluxing in beakers or crucibles: Collaborative study. J. AOAC Int..

[25] SAS (2002). SAS/STAT User’s Guide.

[26] NRC (2000). National Research Council: Nutrient Requirements of Beef Cattle: Update 2000.

[27] Lining K., Shaver R.D., Grant R.J., Schmidt R.J. (2018). Silage review: Interpretation of chemical, microbial, and organoleptic components of silages. J. Dairy Sci..

[28] Park S.J., Beak S.-H., Jung D.J.S., Kim S.Y., Jeong I.H., Piao M.Y., Kang H.J., Fassah D.M., Na S.W., Yoo S.P. (2018). Genetic, management, and nutritional factors affecting intramuscular fat deposition in beef cattle-A review. Asian-Australas. J. Anim. Sci..

[29] Sommart K., Parker D.S., Rowlinson P., Wanapat M. (2000). Fermentation characteristics and microbial protein synthesis in an in vitro system using cassava, rice straw and dried Ruzi grass as substrates. Asian-Australas. J. Anim. Sci..

[30] IPCC (2019). Emissions from Livestock and Manure Management, in 2019 Refinement to the 2006 IPCC Guidelines for National Greenhouse Gas Inventories. https://www.ipcc-nggip.iges.or.jp/public/2019rf/pdf/4_Volume4/19R_V4_Ch10_Livestock.pdf.

[31] Muca E., Buonaiuto G., Lamanna M., Silvestrelli S., Ghiaccio F., Federiconi A., De Matos Vettori J., Colleluori R., Fusaro I., Raspa F. (2023). Reaching a wider audience: Instagram’s role in dairy cow nutrition education and engagement. Animals.

